# Supplementary material for: A Precise Prediction Method for the Properties of API-Containing Tablets Based on Data from Placebo Tablets
Source: Pharmaceutics. 2020 Jun 28;12(7):601. doi: 10.3390/pharmaceutics12070601 (PMC7408303; doi:10.3390/pharmaceutics12070601)
Supplement: Supplementary file 1 [file pharmaceutics-12-00601-s001.pdf]

pharmaceutics

MDPI

Supplementary Materials: A Precise Prediction Method for the Properties of API-Containing Tablets Based on Data from Placebo Tablets

Yoshihiro Hayashi, Kaede Shirotori, Atsushi Kosugi, Shungo Kumada, Kok Hoong Leong, Kotaro Okada and Yoshinori Onuki

Table S1. Tablet properties of API-containing tablets prepared according to extreme vertices design.

Type of API	Rp.	TS (MPa)					DT (s)				
		20% API	30% API	40% API	50% API	60% API	20% API	30% API	40% API	50% API	60% API
ACE	1	2.45	2.16	1.44	1.19	0.76	20.0	19.3	13.7	15.7	28.7
	2	1.38	1.14	1.00	0.76	0.57	30.3	22.7	18.3	18.7	13.3
	2 (duplicates of the centroid)	1.35	0.97	1.02	0.76	0.46	29.3	21.7	17.0	22.3	17.7
	3	1.19	0.93	0.67	0.74	0.49	40.7	34.0	34.3	32.3	28.3
	4	1.03	0.84	0.75	0.47	0.34	15.3	12.7	21.0	15.7	16.3
	5	2.45	2.09	1.76	1.40	0.82	26.7	17.3	14.7	14.7	20.3
	6	1.84	1.40	1.16	0.93	0.52	21.0	15.0	16.3	17.7	16.3
	7	0.75	0.55	0.40	0.40	0.34	53.0	37.7	33.0	28.0	21.3
	8	0.40	0.51	0.20	0.40	0.32	96.3	94.3	79.3	54.3	35.3
	9	2.48	2.05	1.59	1.22	0.72	26.3	16.3	14.3	15.3	13.7
ETZ	1	2.28	2.14	1.92	2.26	2.17	27.3	20.3	21.0	24.7	30.0
	2	1.32	1.30	1.36	1.42	1.61	30.3	23.3	25.7	28.3	37.3
	2 (duplicates of the centroid)	1.34	1.17	1.27	1.54	1.29	33.0	17.0	21.3	33.0	32.3
	3	0.97	0.75	0.75	1.12	1.31	42.7	31.7	27.0	25.7	29.3
	4	1.12	1.07	1.17	1.33	1.12	16.0	17.7	17.7	44.0	75.3
	5	2.49	2.48	2.11	2.27	2.32	37.0	31.3	25.0	39.3	64.3
	6	1.74	1.65	1.59	1.78	1.43	25.7	23.0	24.3	38.3	53.3
	7	0.54	0.64	0.42	1.17	0.94	57.7	34.3	36.0	30.0	41.0
	8	0.33	0.53	0.29	0.71	0.96	93.0	82.0	48.3	54.0	46.0
	9	2.40	2.21	2.02	2.19	2.25	35.3	21.3	21.0	32.7	48.7
NIC	1	2.68	2.50	2.26	2.20	1.81	29.7	27.3	34.3	54.7	52.3
	2	1.44	1.32	1.45	1.27	1.38	23.7	29.3	22.7	29.3	30.0
	2 (duplicates of the centroid)	1.42	1.42	1.32	1.39	1.33	29.7	18.0	24.7	34.7	38.3
	3	1.31	1.03	1.34	1.33	1.35	46.0	30.3	28.3	22.3	24.7

	4	1.19	1.10	1.24	1.15	0.84	17.0	22.3	50.7	147.3	180.7
	5	2.45	2.77	2.87	2.49	2.20	48.3	72.3	133.0	269.7	303.7
	6	1.77	1.95	1.84	1.84	1.39	29.3	43.3	67.7	260.3	232.3
	7	0.66	0.75	0.62	0.78	0.76	43.3	33.7	16.0	22.0	25.0
	8	0.53	0.47	0.44	0.66	0.77	98.0	74.7	57.3	38.0	38.0
	9	2.72	2.57	2.54	2.49	1.99	47.3	37.3	51.0	115.3	220.7
PYH	1	1.99	1.77	1.68	1.44	1.22	43.7	27.0	17.7	26.7	20.3
	2	1.14	1.00	0.99	0.83	0.86	26.0	25.7	12.3	19.0	13.0
	2 (duplicates of the centroid)	1.44	1.07	0.83	0.96	0.64	26.7	13.3	10.7	18.0	10.3
	3	1.31	0.95	0.75	0.82	0.75	44.3	25.3	27.3	24.0	17.3
	4	0.99	0.88	0.79	0.72	0.47	20.7	15.3	16.0	21.7	14.0
	5	2.59	2.16	1.77	1.72	1.42	70.0	57.0	67.3	55.0	37.7
	6	1.50	1.45	1.30	1.20	0.78	26.3	24.3	17.7	33.3	15.0
	7	0.61	0.52	0.38	0.46	0.40	65.3	33.0	33.0	29.0	21.7
	8	0.39	0.37	0.27	0.48	0.38	193.0	115.3	71.3	66.3	30.7
	9	3.03	1.91	1.75	1.59	1.03	113.3	35.7	28.3	42.0	21.0

Table S2. The estimated parameters of regression lines.

**Drug Load**				**20%**				**30%**				**40%**				**50%**				**60%**			
	**Type of API**			**ACE**	**ETZ**	**NA**	**PYH**	**ACE**	**ETZ**	**NA**	**PYH**	**ACE**	**ETZ**	**NA**	**PYH**	**ACE**	**ETZ**	**NA**	**PYH**	**ACE**	**ETZ**	**NA**	**PYH**
Parmeters of equation (2) and (3)	TS		a	0.490	0.517	0.459	0.526	0.378	0.466	0.551	0.427	0.373	0.436	0.581	0.360	0.239	0.374	0.439	0.297	0.120	0.324	0.343	0.249
			b	0.007	−0.082	0.164	-0.028	0.211	0.162	0.030	0.033	−0.095	−0.061	−0.021	−0.020	0.211	0.416	0.310	0.240	0.222	0.704	0.495	0.185
	DT	Orange area	a	0.057	0.105	0.156	0.246	0.023	0.068	0.250	0.208	−0.032	0.037	0.411	0.256	−0.005	−0.023	0.611	0.166	0.020	−0.055	0.614	0.118
			b	14.315	14.113	14.185	16.234	12.247	16.439	17.841	11.590	21.569	17.008	43.269	11.388	15.757	44.419	136.342	18.672	15.974	76.322	169.615	11.874
		Blue area	a	0.007	0.005	0.005	0.032	0.009	0.009	0.009	0.021	0.008	0.003	0.015	0.008	0.001	0.013	0.043	0.012	0.005	-0.008	-0.038	0.004
			b	0.136	0.307	0.388	−1.790	−0.152	−0.313	−0.488	−1.295	−0.240	0.034	−1.668	−0.318	0.379	−1.459	-6.428	−0.886	−0.377	0.582	2.829	−0.331
			c	10.634	8.815	8.444	42.464	12.355	20.371	28.097	31.908	22.780	16.159	75.961	19.071	8.613	65.991	249.179	33.768	21.481	67.386	142.016	18.515
